# Role of UDP-glucose ceramide glucosyltransferase in venous malformation

**DOI:** 10.3389/fcell.2023.1178045

**Published:** 2023-05-19

**Authors:** Sheng Chen, Yuan Wang, Liangliang Kong, Yi Ji, Jie Cui, Weimin Shen

**Affiliations:** Department of Burns and Plastic Surgery, Children’s Hospital of Nanjing Medical University, Nanjing, Jiangsu, China

**Keywords:** venous malformation, UDP-glucose ceramide glucosyltransferase, Akt, mTOR, angiogenesis

## Abstract

Venous malformation (VM) results from the abnormal growth of the vasculature; however, the detailed molecular mechanism remains unclear. As a glycosyltransferase, UDP-glucose ceramide glucosyltransferase (UGCG) is localized to the Golgi body and is a key enzyme in the first step of glycosphingolipid synthesis. Here, we aimed to explore the relationship between UGCG and the development of VM. First, investigations using RT-qPCR and Western blotting on the diseased vasculature of VM patients and normal vascular tissues revealed that UGCG expression was markedly elevated in the diseased vessels. Subsequently, immunofluorescence assay showed that UGCG was co-localized with CD31, an endothelial cell marker, in tissues from patients with VM and healthy subjects. Then, we established TIE2-L914F-mutant human umbilical vein endothelial cells (HUVECs) by lentivirus transfection. Next, Western blotting revealed that UGCG expression was considerably higher in HUVECs^TIE2-L914F^. In addition, we established a UGCG-overexpressing HUVECs line by plasmid transfection. With the CCK8 cell proliferation experiment, wound healing assay, and tube formation assay, we found that UGCG could promote the proliferation, migration, and tube formation activity of HUVECs, whereas the inhibition of UGCG could inhibit the proliferation, migration, and tube formation activity of HUVECs^TIE2-L914F^. Finally, Western blotting revealed that UGCG regulates the AKT/mTOR pathway in HUVECs. These data demonstrated that UGCG can affect the activity of vascular endothelial cells and regulate the AKT/mTOR signaling pathway; this is a potential mechanism underlying VM pathogenesis.

## Introduction

Venous malformation (VM) is the most common vascular malformation, with an incidence rate of 1–5/10,000 (Cooke-Barber et al., 2020). This disease occurs at birth and gradually intensifies with age, hormone level changes, or trauma, which can cause pain, bleeding, anatomical distortion, and organ dysfunction (Rikihisa et al., 2020). VM treatments have been considerably limited, primarily including compression garments, sclerotherapy, and surgical resection (Budge et al., 2021). However, these treatments are often less effective and regrowth is common (Goines et al., 2018). Therefore, there is an urgent need for thorough investigations on VM and the development of new treatment methods to achieve better clinical efficacy.

At present, the pathogenesis of VM is not completely clear. Recent studies have shown that TIE2 and PIK3CA are the two most important genes that cause VM (Nätynki et al., 2015; Castillo et al., 2016; Wang et al., 2021). The researchers found that TIE2 and PIK3CA mutations in VM can induce the constitutive activation of AKT and enhance the activity of the PI3K/AKT/mTOR pathway to promote angiogenesis. mTOR is the direct substrate of AKT kinase, which promotes protein translation, angiogenesis, and metabolism. Inhibiting mTOR can inhibit cell growth and induce apoptosis. Rapamycin (RAPA), an mTOR signaling pathway inhibitor, has recently shown good efficacy in some patients with complex vascular malformations, including VM (Boscolo et al., 2015; Dodds et al., 2020; Harbers et al., 2021). RAPA has been reported to be better than TIE2-TKI (the inhibitor of TIE2) in the treatment of VM murine model (Boscolo et al., 2015). Some researchers believe that this is because RAPA can directly inhibit the AKT/mTOR signaling pathway and bypass TIE2 (Van Damme et al., 2020). Nevertheless, there are still some VM patients with poor efficacy after RAPA treatment, and the underlying mechanism is not completely clear (Geeurickx and Labarque, 2021).

Although TIE2 and PIK3CA mutations explain the occurrence of most sporadic VMs, the pathogenesis of some VMs remains unclear. In recent years, increasing evidence has shown that angiogenesis is closely related to biological metabolism, and the method by which metabolism regulates vascular germination is parallel to the control of genetic signals (De Bock et al., 2013; Li et al., 2019). With the development of metabolomics, ceramide and glycosphingolipid (GSL) metabolism has been increasingly studied in angiogenesis and vascular remodeling (Cogolludo et al., 2019; Andersson et al., 2021; McGurk et al., 2021). The only enzyme in the GSL metabolic pathway that can produce glucose ceramide from scratch is UDP-glucose ceramide glucosyltransferase (UGCG) (Wegner et al., 2018). It binds to ceramide with a β-Glycoside bond to form glucose ceramide, which makes cells escape the pro-apoptotic effect of ceramide (Li et al., 2021). The increased expression of UGCG has been proven to promote the activation of the AKT/mTOR signaling pathway in various cell lines (Natoli et al., 2010; Hartwig and Höglinger, 2021). In studies on emphysema, UGCG has been proven to affect the apoptosis of pulmonary microvascular endothelial cells by regulating the mTOR pathway (Koike et al., 2019). Furthermore, UGCG is also associated with Andeson-Fabry disease (AFD), a rare X-linked recessive inherited congenital error of sphingolipid metabolism, often manifested as systemic vasokeratinoma formation, similar to a partial VM phenotype (Tuttolomondo et al., 2021; Matarneh et al., 2022). However, the role of UGCG in VM is unclear. Therefore, we assessed UGCG expression levels in VMs and explored the potential significance.

## Materials and methods

### Patients and samples

Twenty-four patients with VM (no history of preoperative treatment) from 2020 to 2021 in the Children’s Hospital of Nanjing Medical University (Nanjing, China) were selected, and surgically excised VM tissues were collected (Table 1). Twenty cases of normal tissues (Vascular tissues from trauma patients) were selected as the control group. The tissue specimens were transferred to liquid nitrogen for preservation. This study was approved by the Ethics Committee of the Nanjing Medical University (201902068-1), and written informed consent was obtained from all patients or their guardians before specimen collection.

**TABLE 1:** Clinical characteristics of patients.

Id	Gender	Age (year)	Location	Pathological classification
1	Male	10	Leg	VM
2	Female	5	Hand	VM
3	Female	2	Haunch	VM
4	Female	4	Hand	VM
5	Female	1	Face	VM
6	Male	2	Hand	VM
7	Female	3	Back	VM
8	Female	9	Face	VM
9	Male	4	Hand	VM
10	Male	6	Foot	VM
11	Male	6	Face	VM
12	Male	4	Foot	VM
13	Female	3	Chest	VM
14	Female	1	Foot	VM
15	Male	5	Neck	VM
16	Female	5	Face	VM
17	Male	10	Lip	VM
18	Male	6	Chest	VM
19	Male	3	Tongue	VM
20	Female	10	Leg	VM
21	Female	5	Hand	VM
22	Female	8	Leg	VM
23	Female	4	Chest	VM
24	Male	2	Foot	VM
VM, venous malformation				

### Cell culture and treatment

HUVECs (cat. no. 8000) were purchased from ScienCell (United States) and cultured in endothelial cell medium (ECM; ScienCell Research Laboratories, Inc., San Diego, CA, United States) containing 10% fetal bovine serum (FBS). Cells were cultured in a humidified atmosphere with 5% CO2 at 37°C. The culture medium was replaced every 2–3 days according to the condition of cell growth. The cells were subcultured at approximately 80%–90% confluence. For all experiments, HUVECs from passages 2 to 9 were used.

### Cells transduction

Lentiviral vectors expressing TIE-WT and TIE2-L914F were generated by GenePharma (Shanghai, China). Briefly, full-length TIE2-WT or TIE2-L914F were cloned into pMXs vector and packaging cell line 293-GPG VSV-G was transfected for retrovirus production with Fugene 6 (Roche). HUVECs were seeded on a 6-well plate overnight and then transduced with lentiviral vectors expressing TIE-WT and TIE2-L914F in the presence of polybrene (8 mg/mL). After transduction overnight, puromycin was used to screen the positive cells. The transduction efficiency was confirmed by fluorescence microscopy and Western blotting. The UGCG expression plasmid (pEX-3 vector) and the blank vector were purchased from GenePharma (Shanghai, China). Stable transduction was performed using Lipofectamine^®^ 2000 Reagent (Invitrogen by Life Technologies) according to the manufacturer’s protocol. Cells were incubated at 37°C with 5% CO_2_ for 5–6 h and cultured in a complete medium for 24–48 h for the following experiment.

### Wound healing assay

HUVECs were seeded at a density of 1 × 10^6^ cells per well in 6-well plates. After cells were attached to the bottom, wounds were generated by a sterile pipette aspiration head and the ECM was replaced with a medium without FBS. Images from the same perspective were observed and collected every 24 h.

### Cell count kit-8

This experiment was performed using the cell count kit-8 (CCK8) (Beyotime Institute of Biotechnology). After cell starvation overnight, HUVECs (3,000/well) were placed on 96-well plates, and CCK8 (10 μL) was added to each well. After 2 h of incubation, the absorbance values were measured at 450 nm, using a Multiskan FC (Thermo Fisher Scientific, Inc.).

### *In vitro* tube formation assay

Tube formation assay was performed as described in previous studies. It was performed in 96-well plates coated with 50 μL of Matrigel Basement Membrane Matrix Growth Factor Reduced (cat. 354230; Corning Inc.), which was allowed to form a gel at 37°C for 1 h. Thereafter, 3 × 10^4^ cells in ECM without FBS were seeded onto the Matrigel and incubated at 37°C for 12 h. The formation of tubular structures was captured using a Nikon Eclipse 80i Microscope (Nikon, Japan). Vascular lengths and branch points in each group were quantified using the ImageJ software plin-in Angiogenesis Analyzer.

### Immunofluorescence

Tissue samples were fixed in 4% paraformaldehyde and embedded in paraffin. The tissue sections were cut into 4-mm thick sections on a slicer. Antigen repair was performed with Tris/EDTA buffer (pH 9.0). The samples were blocked with 5% bovine serum albumin and incubated with primary antibodies against UGCG (1:200; cat. no.sc-293235; Santa Cruz Biotechnology, Inc.) and CD31 (1:200; cat. no.3528; Cell Signaling Technology, Inc.) at 4°C overnight. The samples were incubated with solutions containing secondary antibodies conjugated to Cy3 (1:200; cat. no.A0516; Beyotime Institute of Biotechnology) and FITC (1:200; cat. no.A0568; Beyotime Institute of Biotechnology) at room temperature for 1 h. DAPI (1:1000; Beyotime Institute of Biotechnology) was used for nuclear counterstaining. Sample analysis and image acquisition were carried out using a laser confocal microscope (Zeiss, Germany).

### Western blot analysis

First, the tissue or cells were washed twice with ice-cold PBS and incubated for 30 min on ice with RIPA buffer (Beyotime Institute of Biotechnology). Total protein was collected after high-speed centrifugation (10,000 g) for 30 min at 4°C, and the protein concentration was detected using a BCA kit (Beyotime Institute of Biotechnology). Subsequently, the lysate was loaded into 7.5% SDS-PAGE and transferred to 0.22 µm polyvinylidene difluoride membranes. Membranes were blocked with skimmed milk powder of 5% mass concentration at room temperature for 1.5 h and incubated with the following primary antibodies: GAPDH (1:1,000; cat. no.10494-1-AP; ProteinTech Group, Inc.), β-actin (1:1,000; cat. no.66009-1-Ig; ProteinTech Group, Inc.), vinculin (1:1,000; cat. no.66305-1-Ig; ProteinTech Group, Inc.), UGCG (1:1,000; cat. no.sc-293235; Santa Cruz Biotechnology, Inc.), AKT (1:1,000; cat. no.2920; Cell Signaling Technology, Inc.), p-AKT (1:1,000; cat. no.4060; Cell Signaling Technology, Inc.), mTOR (1:1,000; cat. no.66888-1-Ig; ProteinTech Group, Inc.), p-mTOR (1:1,000; cat. no.67778-1-Ig; ProteinTech Group, Inc.), TIE2 (1:1,000; cat. no.ab221154; Abcam), p-TIE2 (1:1,000; cat. no.ab151704; Abcam) and Cyclin D1 (1:1,000; cat. no.60186-1-Ig; ProteinTech Group, Inc.) at 4°C overnight. Samples were washed three times with TBST. The membrane and HRP-labeled goat anti-secondary antibody (1:2,000; cat. no.SA00001-1 or cat. no.SA00001-2; ProteinTech Group, Inc.) were incubated at room temperature for 1 h. Grayscale analysis was performed with ImageJ software.

### Real-time quantitative reverse transcription PCR analysis

Tissue samples from patients were stored in liquid nitrogen until use. Total RNA from cultured cells and tissues was extracted with TRIzol reagent (Vazyme Biotech Co., Ltd.), and cDNA was synthesized using the PrimeScript RT reagent kit (Takara Biotechnology Co., Ltd.) according to the manufacturer’s instructions. qPCR was conducted using the SYBR Green PCR master mix (Takara Biotechnology Co., Ltd.) in 20 μL reactions according to the manufacturer’s instructions. The reaction protocol included incubations at 95°C for 30 s, followed by 40 cycles of 95°C for 5 s and 60°C for 30 s. GAPDH served as an endogenous control. Relative expression was calculated using the comparative quantification cycle (Cq) method (2^−ΔΔCq^). The primer sequences used for real-time PCR are listed as follows.

$$UGCG-F:5^{\prime }GAATGGCCGTCTTCGGGTT3^{\prime }$$

$$UGCG-R:5^{\prime }AGGTGTAATCGGGTGTAGATGAT3^{\prime }$$

$$GAPDH-F:5^{\prime }GCACCGTCAAGGCTGAGAAC3^{\prime }$$

$$GAPDH-R:5^{\prime }GGATCTCGCTCCTGGAAGATG3^{\prime }$$

### Statistical analysis

All experiments were repeated ≥3 times and data are presented as mean ± standard deviation. All data were analyzed using SPSS 23.0 software (IBM Corp., Armonk, NY, United States). The differences between two means were analyzed with the Student’s *t*-test. Comparisons among ≥3 groups were performed using one-way analysis of variance (ANOVA) followed by Tukey’s multiple comparisons. Values of *p* < 0.05 were statistically significant. Data were visualized with GraphPad Prism 8.0 software (GraphPad Software, La Jolla, CA, United States).

## Results

### Upregulation of UGCG expression in patients with VM

**FIGURE 1:**
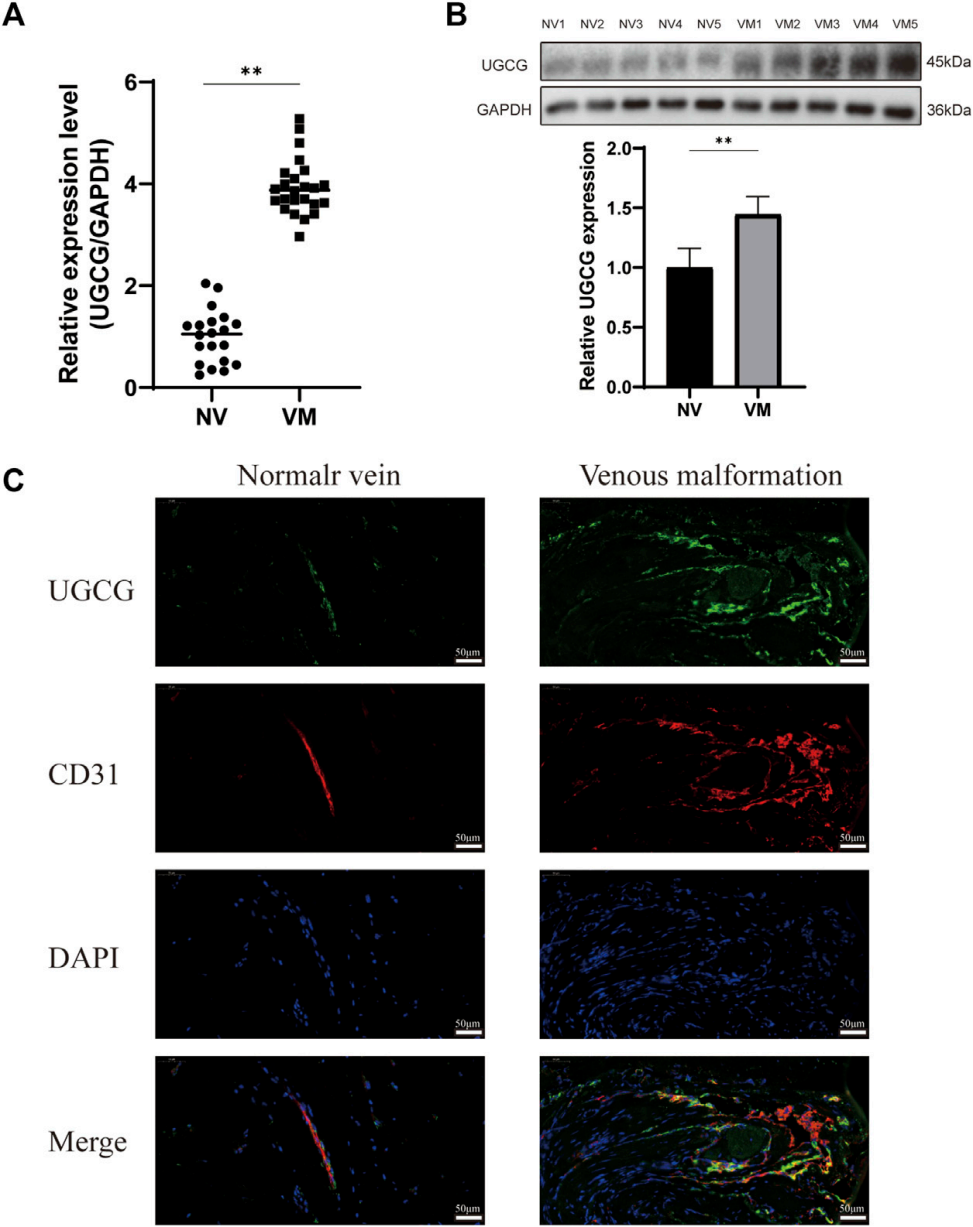
Upregulation of UDP-glucose ceramide glucosyltransferase (UGCG) expression in patients with venous malformation. **(A,B)** The mRNA **(A)** and protein **(B)** expression levels of UGCG were detected in the vascular tissues of venous malformation patients and normal subjects. **(C)** UGCG and CD31 were co-stained in the tissue sections from venous malformation patients and normal subjects. DAPI was used to indicate the cell nucleus. **p < 0.01 as compared with the normal vein. Scale bar, 50 mm.

**FIGURE 2:**
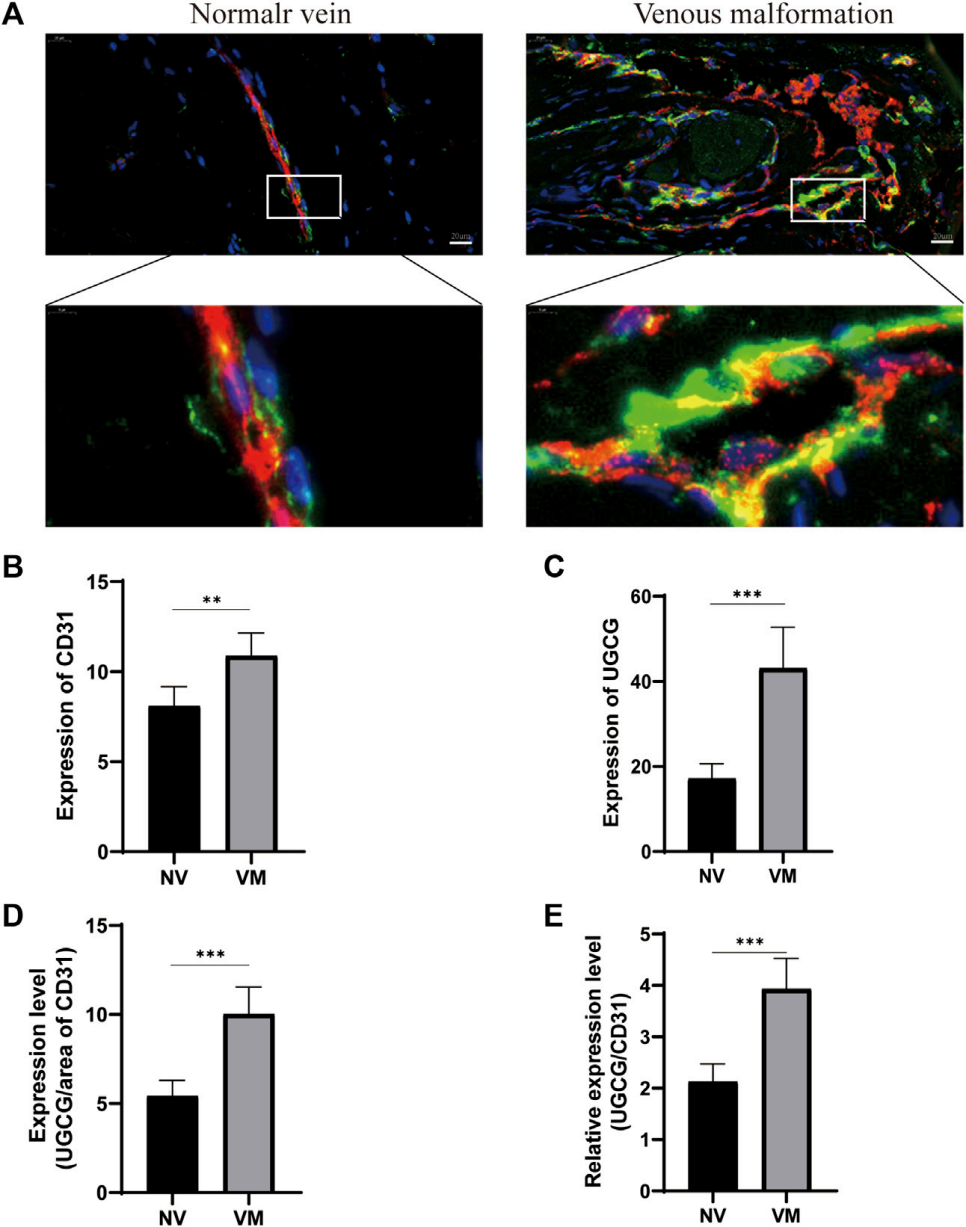
Upregulation of UGCG and CD31 expression in patients with venous malformation. **(A)** UGCG and CD31 were co-stained in the tissue sections from venous malformation patients and normal subjects. DAPI was used to indicate the cell nucleus. **(B,C)** Expression levels of UGCG and CD31 were detected in the vascular tissues of venous malformation patients and normal subjects. **(D,E)** Expression of UGCG normalized to the area and expression level of CD31. **p < 0.01, ***p < 0.001 as compared with the normal vein. Scale bar, 20 mm.

Preliminary literature study indicated a possible link between UGCG and VM (Rajesh et al., 2005; Sasaki and Toyoda, 2013; Wegner et al., 2018; Kang et al., 2019; Koike et al., 2019). Therefore, we speculate that UGCG may play a role in VM progression. Twenty-four patients were included in this study. First, we determined the level of UGCG expression in the vascular tissues of VM patients and healthy subjects using RTq-PCR. We discovered that the mRNA level of UGCG in patients with VM was considerably greater than that in the control group (Figure 1A). Next, Western blotting results indicated that UGCG protein levels were considerably higher in the vascular tissues of patients with VM (Figure 1B). In addition, immunofluorescence revealed that UGCG was co-localized with CD31, an endothelial cell marker, in the tissues from VM patients and the control group (Figure 1C). Given the high vascularity of VM tissues, we locally enlarged the Figures and found that the fluorescence intensity of UGCG in VM tissues was significantly enhanced compared to the control group (Figure 2A). To check, we have calculated the expression area and level of CD31 in the vascular tissues of VM patients and healthy subjects with the results of immunofluorescence. The results showed that the expression level of CD31 were higher in the VM tissues (Figure 2B). Subsequently, the expression level of UGCG was calculated, and normalized to the expression area and level of CD31. The results confirmed that UGCG was significantly increased in VM tissues (Figures 2C–E).

### Upregulation of UGCG expression in TIE2-L914F-mutant HUVECs

**FIGURE 3:**
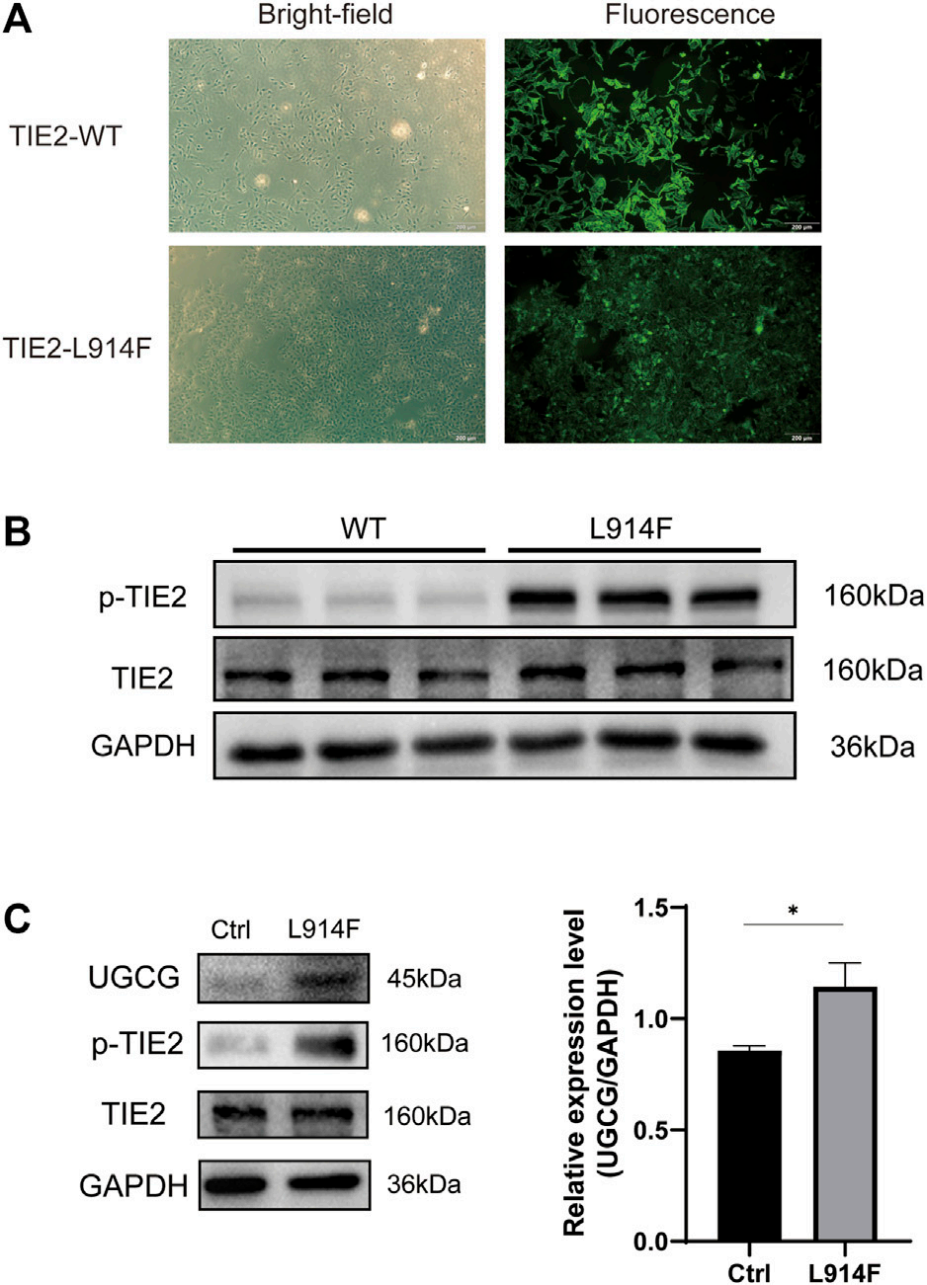
Upregulation of UDP-glucose ceramide glucosyltransferase (UGCG) expression in HUVECs^TIE2-L914F^. **(A)** The bright-field and fluorescent view of HUVECs transduced with TIE2-L914F mutant-GFP lentivirus under the microscope. **(B)** The phosphorylated TIE2 and TIE2 level was detected by WB. **(C)** The UGCG, phosphorylated TIE2 and TIE2 level was detected by WB. *p < 0.05 as compared with the control group. Scale bar, 100 mm. *N* = 3.

Considering that the somatic mutation of TIE2-L914F accounts for 77% of patients with mutation-positive lesions, we transduced HUVECs with TIE2-L914F-mutant GFP lentivirus (Limaye et al., 2009; Soblet et al., 2013). The transfection efficiency was verified by fluorescence microscopy and Western blotting (Figures 3A, B). Then, the expression level of UGCG in HUVECs and HUVECs^TIE2-L914F^ was determined by Western blotting. The results showed that UGCG expression was enhanced in HUVECs^TIE2-L914F^ (Figure 3C).

### Establishing a UGCG-overexpressing HUVECs line

**FIGURE 4:**
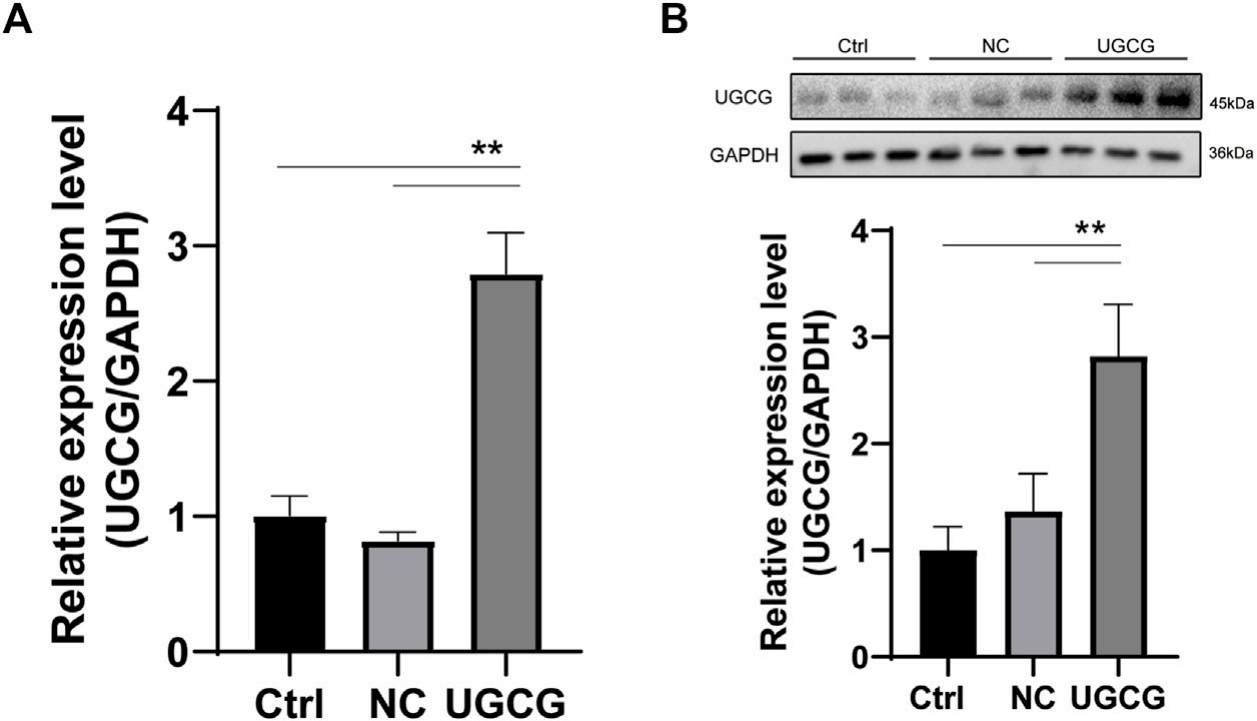
UDP-glucose ceramide glucosyltransferase (UGCG) overexpression in human umbilical vein endothelial cells (HUVECs). **(A)** Expression analysis of UGCG mRNA in HUVECs by RTq-PCR. **(B)** Expression analysis of UGCG protein in HUVECs by WB. **p < 0.01 as compared with the control group. *N* = 3.

HUVECs were transduced with a UGCG expression plasmid (HUVECs/UGCG) or a control vector (HUVECs/NC) (HUVECs/Ctrl = no transfection). The expression levels of UGCG mRNA and protein in transduced HUVECs were detected by RTq-PCR and Western blotting. The results showed that the mRNA and protein expression levels of UGCG in HUVECs/UGCG were significantly higher than those in HUVECs/NC and HUVECs/Ctrl (Figures 4A, B).

### UGCG promotes the proliferation of HUVECs

**FIGURE 5:**
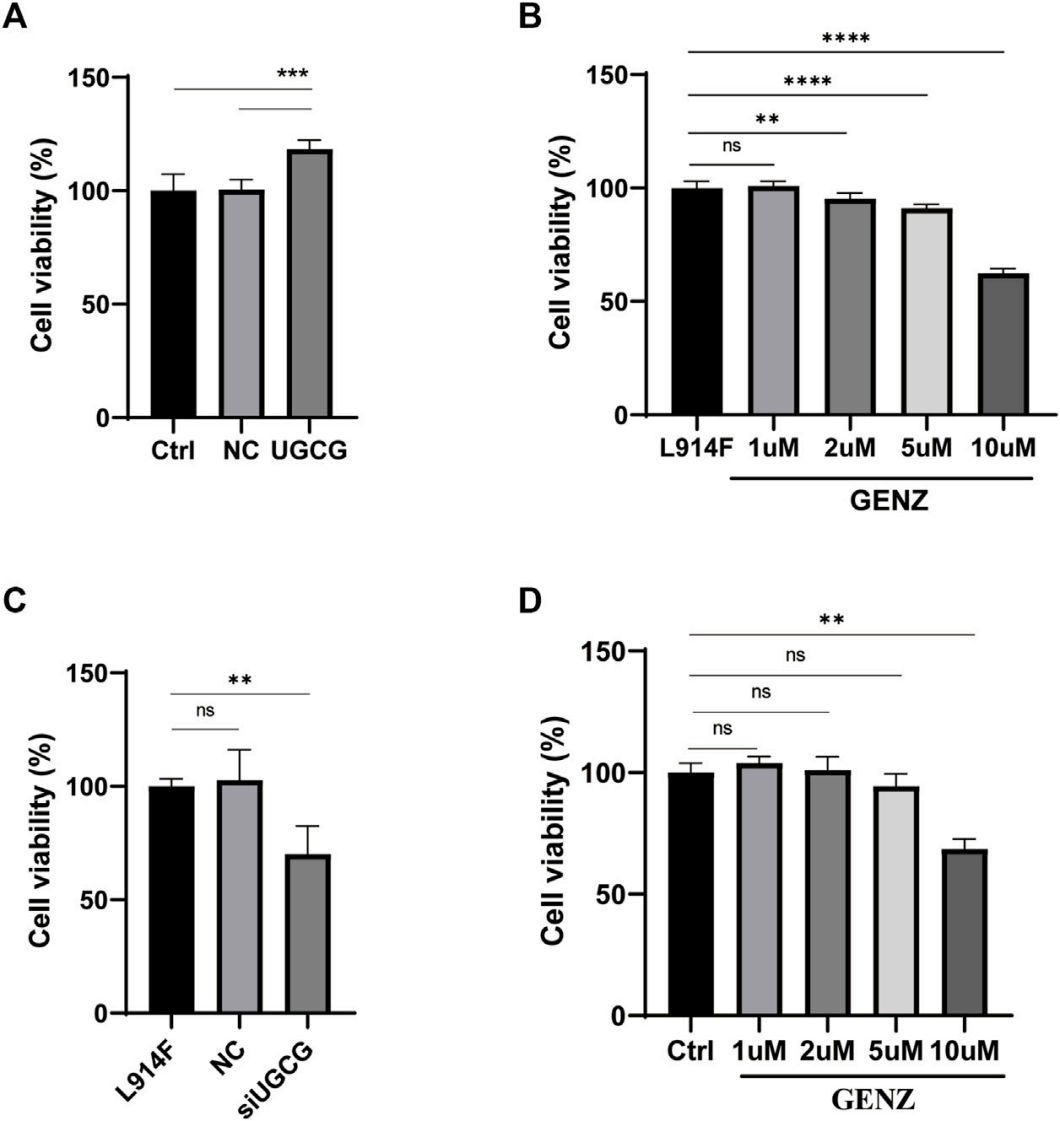
The proliferation of human umbilical vein endothelial cells (HUVECs). **(A)** The absorbance value of HUVECs/Ctrl, HUVECs/NC, and HUVECs/UGCG. **(B)** The absorbance value of HUVECs^TIE2-L914F^ treated with Genz (0, 1, 2, 5, or 10 μM) for 24 h. **(C)** The absorbance value of HUVECs^TIE2-L914F^, HUVECs^TIE2-L914F^/NC, and HUVECs^TIE2-L914F^/siUGCG. **(D) **The absorbance value of HUVECs/Ctrl treated with Genz (0, 1, 2, 5, or 10 μM) for 24 h as compared with the control group, **p < 0.01, ***p < 0.001, ****p < 0.0001 as compared with the control group or HUVECs^TIE2-L914F^. N = 6.

To verify the effect of UGCG on cell proliferation, we used the CCK8 experiment to detect the proliferation of HUVECs after plasmid transfection. The results showed that the cell viability of HUVECs/UGCG was significantly higher than those of HUVECs/Ctrl and HUVECs/NC (Figure 5A). Genz-123346 (Genz; UGCG inhibitor) has been demonstrated to selectively inhibit UGCG in several cellular and animal models (Zhao et al., 2007; Natoli et al., 2010; Chai et al., 2011). To determine the optimal concentration of Genz in HUVECs experiments, we treated HUVECs^TIE2-L914F^ with Genz (1, 2, 5, or 10 μM) for 24 h. The CCK8 experiment showed a significant reduction in cell viability (*p* < 0.01) at the lowest Genz concentration (2 μM) (Figure 5B). In order to test the on-target efficacy of Genz, we conducted CCK8 experiment on siRNA depletion of UGCG in HUVECs^TIE2-L914F^ and Genz exposure of normal HUVECs. The results showed that the cell viability of HUVECs^TIE2-L914F^/siUGCG was significantly lower than those of HUVECs^TIE2-L914F^ and HUVECs^TIE2-L914F^/NC (Figure 5C). In addition, the CCK8 experiment showed a significant reduction in cell viability of normal HUVECs at the lowest Genz concentration (10 μM) (Figure 5D).

### UGCG promotes the migratory ability of HUVECs

**FIGURE 6:**
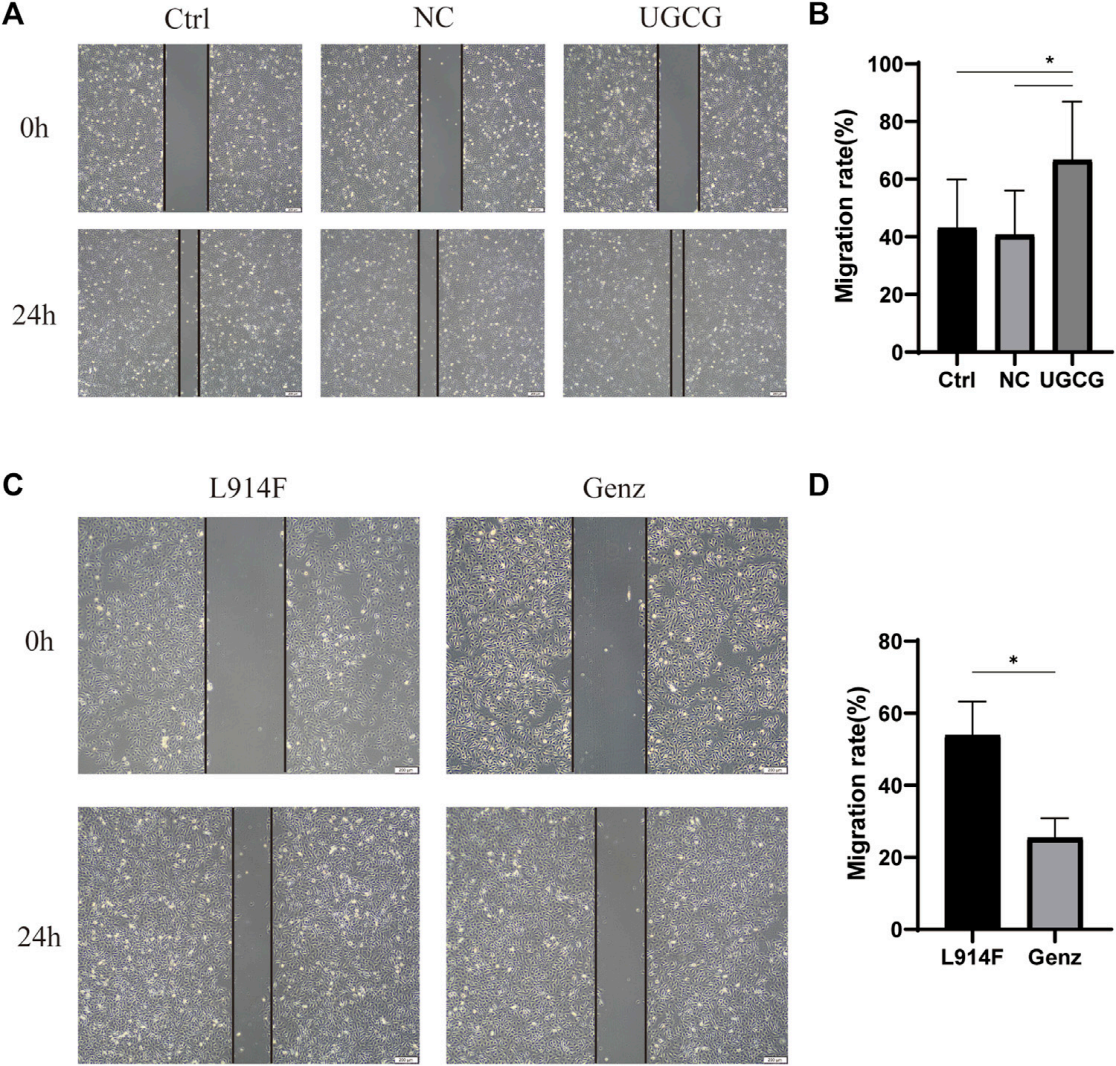
Impaired cell migration in human umbilical vein endothelial cells (HUVECs) **(A)** Migration of HUVECs/Ctrl, HUVECs/NC, and HUVECs/UGCG was performed with an *in vitro* scratch assay. **(C)** Migration of HUVECs^TIE2-L914F^ and HUVECs^TIE2-L914F^ treated with Genz was performed with an *in vitro* scratch assay. **(B,D)** The migration rate of cells was measured and quantified with ImageJ. Images were acquired at 0 h and 24 h in the assay. The lines define the area lacking cells. *p < 0.05 as compared with the control group or HUVECs^TIE2-L914F^. Scale bar, 200 mm. N = 3.

**FIGURE 7:**
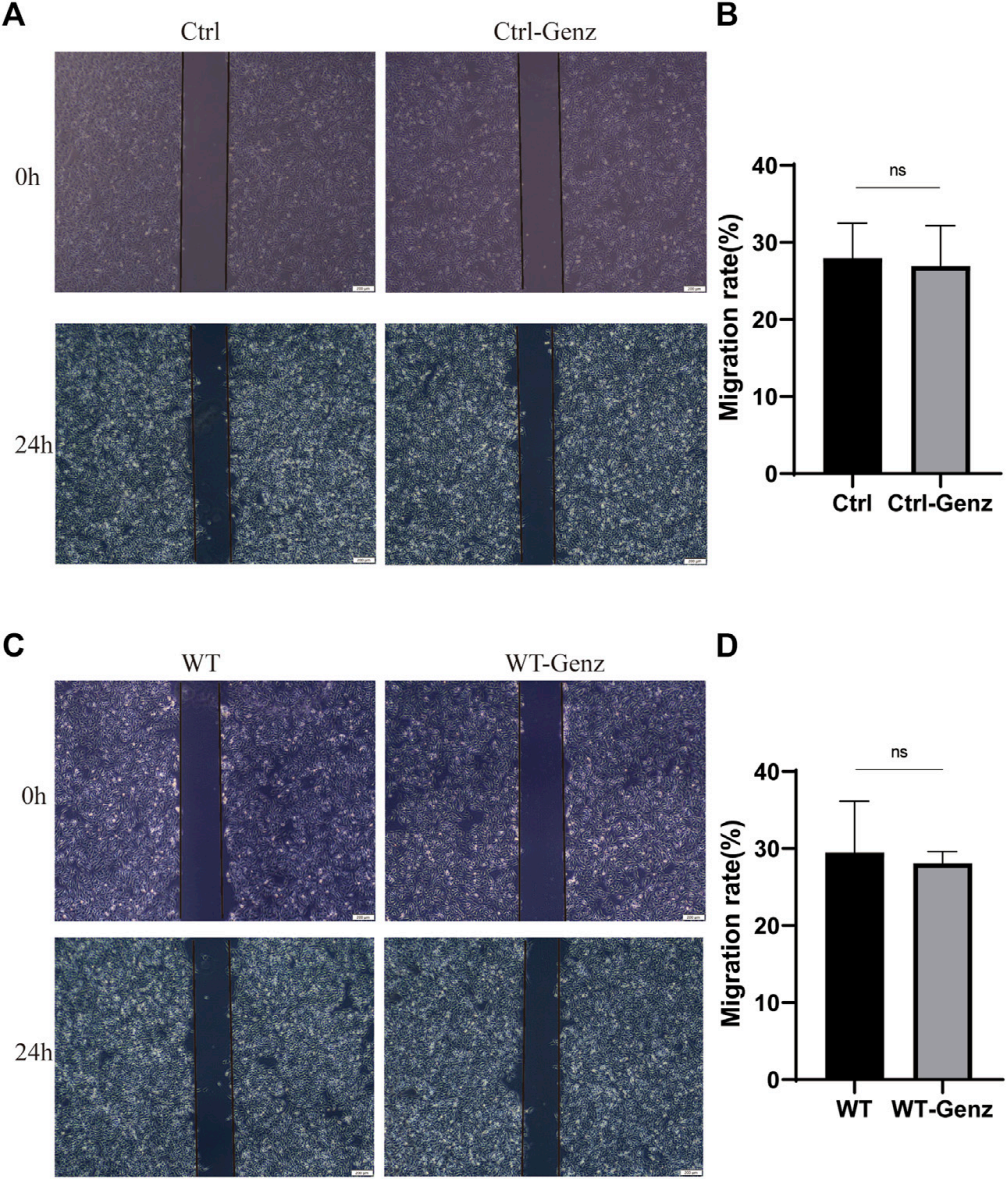
Impaired cell migration in human umbilical vein endothelial cells (HUVECs). **(A)** Migration of HUVECs/Ctrl and HUVECs/Ctrl treated with Genz was performed with an *in vitro* scratch assay. **(C)** Migration of HUVECs^TIE2−WT^ and HUVECs^TIE2−WT^ treated with Genz was performed with an *in vitro* scratch assay. **(B,D)** The migration rate of cells was measured and quantified with ImageJ. Images were acquired at 0 h and 24 h in the assay. The lines define the area lacking cells. Scale bar, 200 mm. N = 3.

We used a wound-healing assay to detect the effect of UGCG on cell migration. The results showed that the number of cells migrating to the scratch midline of HUVECs/UGCG increased significantly, compared with the HUVECs/Ctrl and HUVECs/NC (Figures 6A, B). In addition, cell migration significantly decreased in the HUVECs^TIE2-L914F^ treated with Genz (1 μM), compared with the control group (Figures 6C, D). Besides, we supplemented the wound-healing assay to detect the effect of Genz in HUVECs and HUVECs^TIE2−WT^, and the results showed that Genz had no significant effect on the migratory ability of them (Figures 7A–D).

### UGCG facilitates the tube formation activity of HUVECs

**FIGURE 8:**
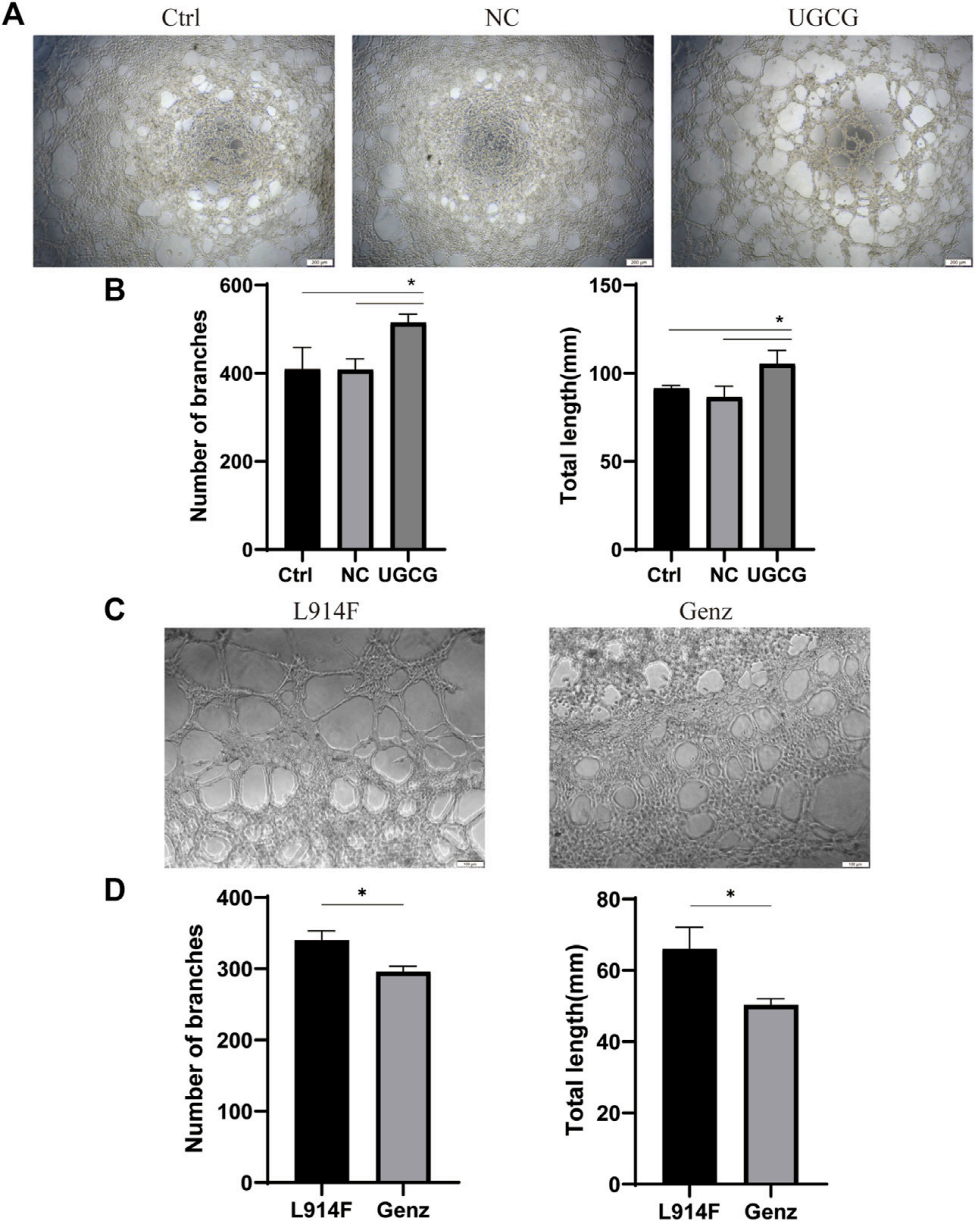
Tube formation activity of human umbilical vein endothelial cells (HUVECs). **(A)** Tube formation of HUVECs/Ctrl, HUVECs/NC, and HUVECs/UGCG was performed with Matrigel. **(C)** Tube formation of HUVECs^TIE2-L914F^ and HUVECs^TIE2-L914F^ treated with Genz was performed with Matrigel. **(B,D)** The number of tube-like structures and the lengths of the branches were measured and quantified with ImageJ. The images were captured after 12 h of incubation. *p < 0.05 as compared with the control group or HUVECs^TIE2-L914F^.Scale bar, 200 mm. N = 3.

**FIGURE 9:**
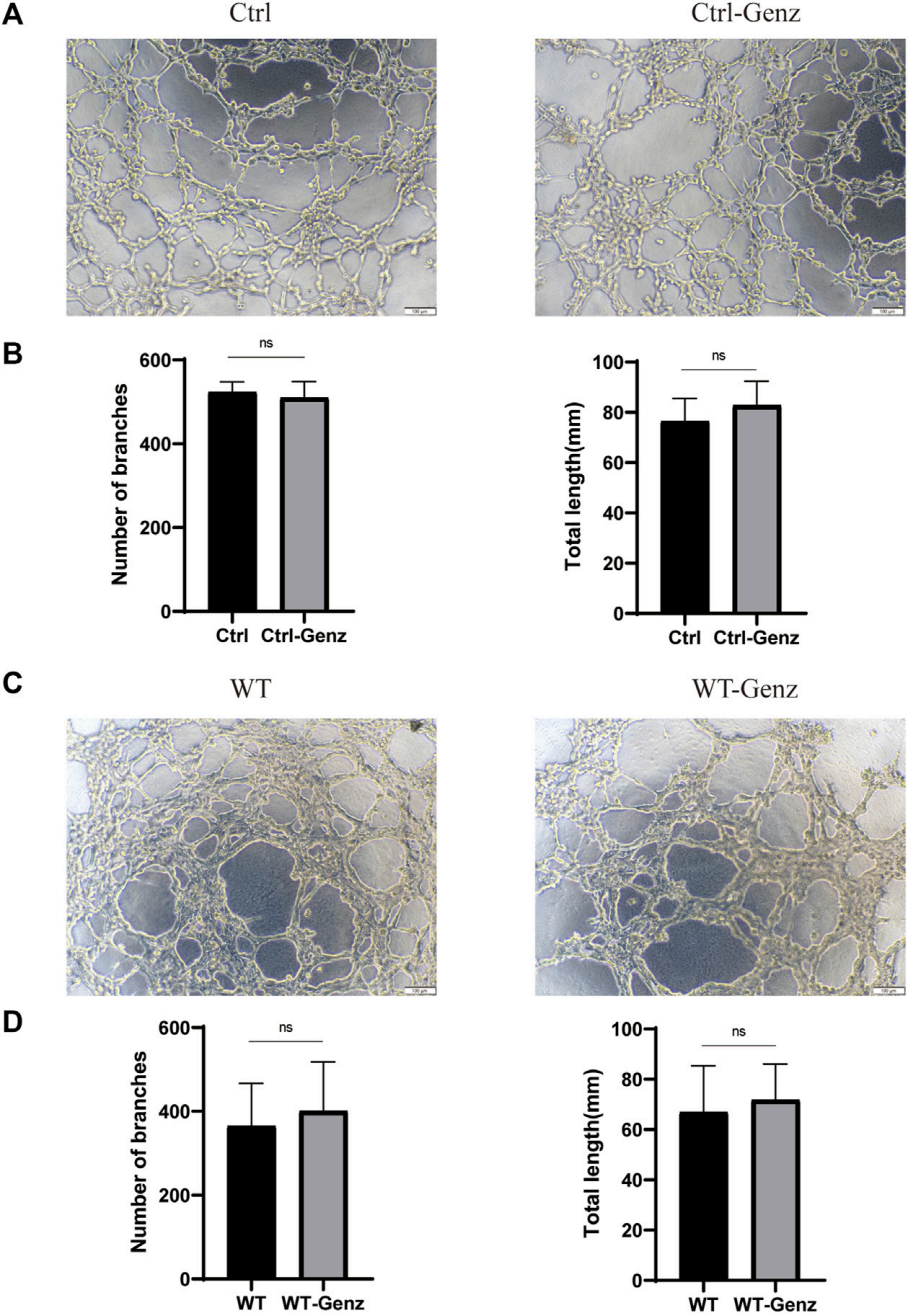
Tube formation activity of human umbilical vein endothelial cells (HUVECs). **(A)** Tube formation of HUVECs/Ctrl and HUVECs/Ctrl treated with Genz was performed with Matrigel. **(C)** Tube formation of HUVECs^TIE2−WT^ and HUVECs^TIE2−WT^ treated with Genz was performed with Matrigel. **(B,D)** The number of tube-like structures and the lengths of the branches were measured and quantified with ImageJ. Scale bar, 200 mm. N = 6.

To validate the effect of UGCG on the tube capacity of HUVECs, we plated HUVECs onto a solubilized and subsequently solidified basement membrane extract matrix. After 12 h of incubation, we determined the mean lumen length and the average number of branches in each group. The results showed that tube formation increased in HUVECs/UGCG, compared with HUVECs/Ctrl and HUVECs/NC (Figures 8A, B). In addition, tube formation decreased in the HUVECs^TIE2-L914F^ treated with Genz (1 μM), compared with the control group (Figures 8C, D). Besides, we supplemented the tube formation experiments to detect the effect of Genz in HUVECs and HUVECs^TIE2−WT^, and the results showed that Genz had no significant effect on the tube formation activity of them (Figures 9A–D).

### UGCG activates the AKT/mTOR signaling pathway in HUVECs

**FIGURE 10:**
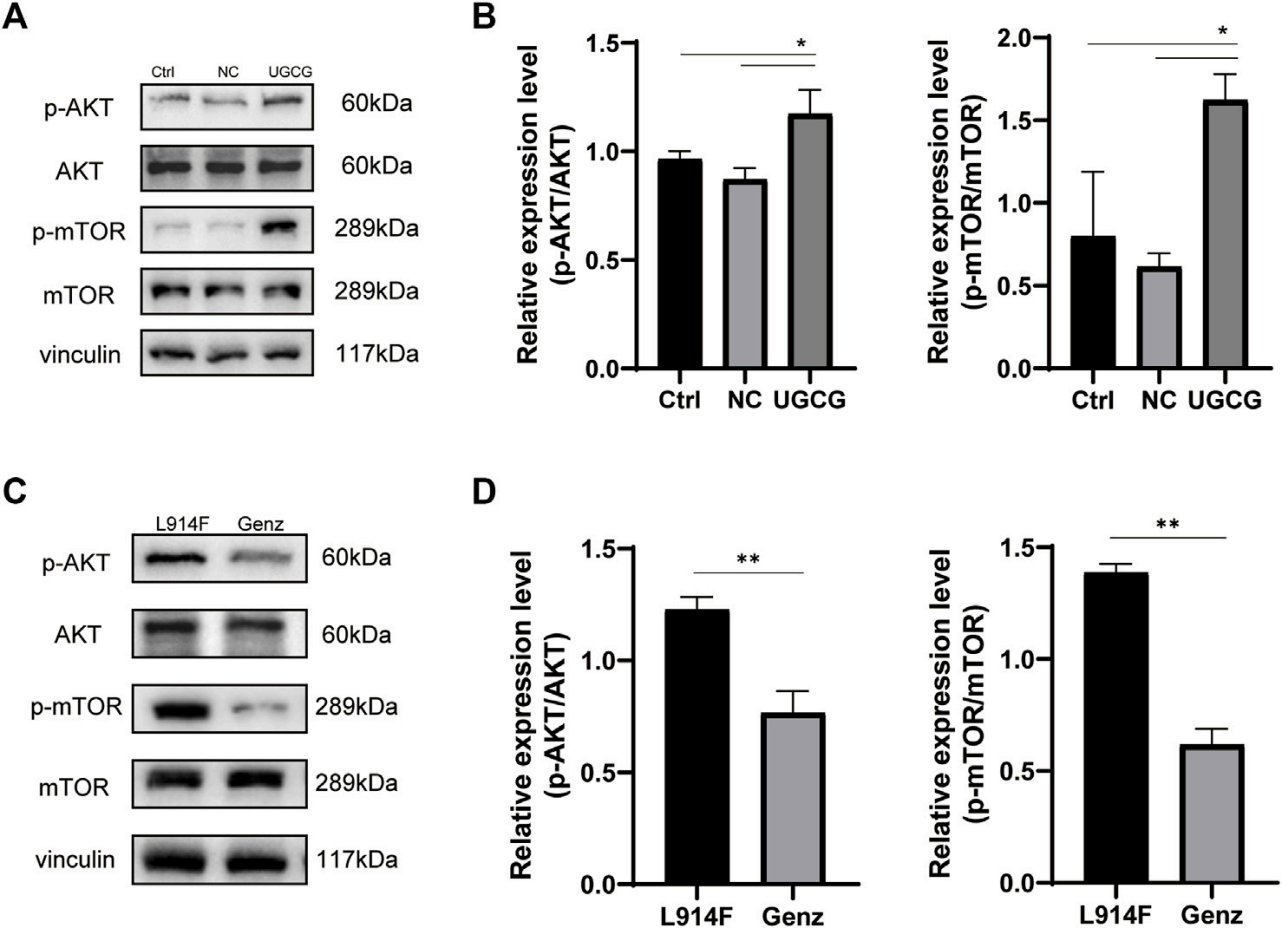
UDP-glucose ceramide glucosyltransferase (UGCG) regulates the AKT/mTOR signaling pathway in human umbilical vein endothelial cells (HUVECs). **(A,B)** Western blot analysis of AKT, p-AKT, mTOR, and p-mTOR on HUVECs/Ctrl, HUVECs/NC, and HUVECs/UGCG. **(C,D)** Western blot analysis of AKT, p-AKT, mTOR, and p-mTOR on HUVECs^TIE2-L914F^ and HUVECs^TIE2-L914F^ treated with Genz. *p < 0.05 as compared with the control group. **p < 0.05 as compared with HUVECs^TIE2-L914F^. N = 3.

**FIGURE 11:**
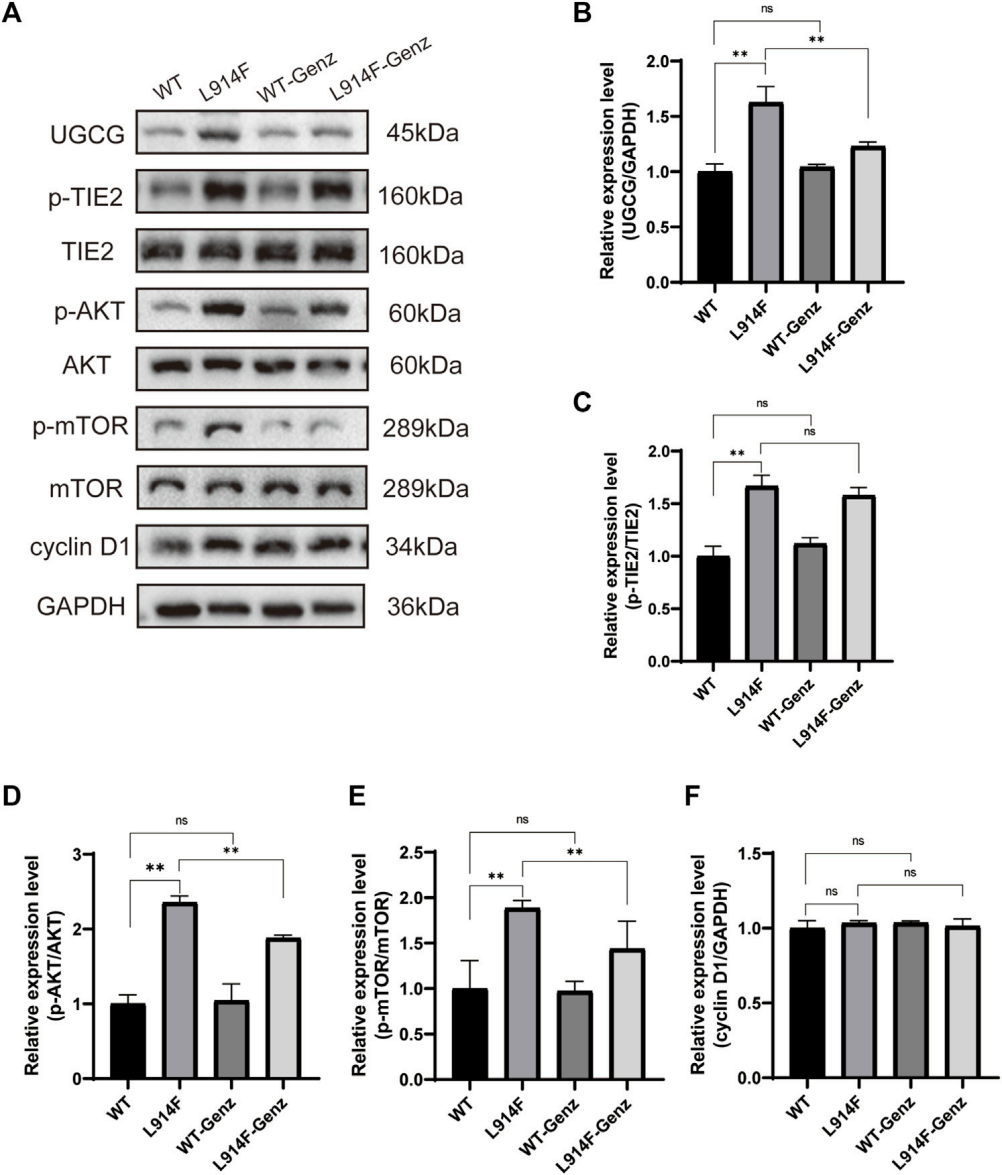
UDP-glucose ceramide glucosyltransferase (UGCG) regulates the AKT/mTOR signaling pathway in human umbilical vein endothelial cells (HUVECs). **(A–F)** Western blot analysis of UGCG, p-TIE2, TIE2, p-AKT, AKT, p-mTOR, mTOR, and Cyclin D1 on HUVECs^TIE2−WT^, HUVECs^TIE2−WT^ treated with Genz, HUVECs^TIE2-L914F^ and HUVECs^TIE2-L914F^ treated with Genz. **p < 0.05 as compared with the HUVECs^TIE2−WT^ or HUVECs^TIE2-L914F^. N = 3.

To further understand how UGCG controls the fate of HUVECs, we determined the effect of UGCG on AKT/mTOR signaling. First, we assessed the phosphorylation of AKT and mTOR in HUVECs/Ctrl, HUVECs/NC, and HUVECs/UGCG by Western blotting. The results showed that the phosphorylation of AKT and mTOR increased after UGCG overexpression (Figures 10A, B). Subsequently, the phosphorylation of AKT and mTOR in HUVECs^TIE2-L914F^, with or without Genz (1 μM) intervention, was detected by Western blotting. The results showed that the expression of p-AKT and p-mTOR in HUVECs^TIE2-L914F^ after Genz intervention significantly decreased, compared with the control group (Figures 10C, D). In addition, we assessed the expression of UGCG, p-TIE2, TIE2, p-AKT, AKT, p-mTOR, and mTOR by Western blotting in HUVECs^TIE2−WT^ and HUVECs^TIE2-L914F^, with or without Genz (1 μM) intervention. The result shows that the expression of UGCG, p-AKT and p-mTOR in HUVECs^TIE2-L914F^ after Genz intervention significantly decreased, compared with the control group, while there was no significant change in HUVECs^TIE2−WT^ (Figure 11A, B, D, E). Besides, there was no significant change in the expression level of p-TIE2 after Genz treatment (Figures 11A, C). Given the direct impact of Genz on cell cycle mechanisms previously confirmed in mouse models (Natoli et al., 2010), we detected the expression of Cyclin D1 by Western blotting, while the expression level of Cyclin D1 did not show significant changes (Figures 11A, F). These data suggest that UGCG can regulate AKT/mTOR signaling in HUVECs.

## Discussion

The most important findings of this study are the increased UGCG expression levels in the diseased vascular tissues of VM patients and HUVECs with TIE2-L914F mutation. Subsequently, we demonstrated in HUVECs and HUVECs^TIE2-L914F^ that UGCG regulates the AKT/mTOR pathway and plays an important role in the proliferation, migration, and tube formation of vascular endothelial cells. In the present study, we demonstrated that UGCG may lead to the pathogenesis of VM by modulating the AKT/mTOR signaling pathway to alter the angiogenic activity of endothelial cells.

In recent years, increasing evidence has shown that the transition of endothelial cells from a long-term static state to an active growth state is not only controlled by genetic signals but also closely related to biological metabolism (Goines et al., 2018). Recently, there has been an increase in the number of studies on sphingolipid metabolism and angiogenesis. The primary enzyme in the first stage of GSL production is UGCG, a glycosyltransferase mostly found on the Golgi apparatus (Schömel et al., 2020a). According to earlier research, overexpression of UGCG can change the metabolic state of cells from quiescent to active and greatly raise the amount of oxidative phosphorylation (Schömel et al., 2020b). The primary job of UGCG is to encourage the formation of glucosylceramide from the glycosylation of ceramide, which results in a range of more complicated GSLs. One of the risk factors for cardiovascular disease is ceramide, a precursor to GSLs. It has been demonstrated that ceramide inhibits angiogenesis by lowering vascular endothelial growth factor production (Bansode et al., 2011). GSLs, which are a part of cell membranes, are crucial in angiogenesis, which is linked to coronary heart disease, vascular problems associated with diabetes, inflammatory vascular disease, and tumor metastasis. Lactoceramide formed by adding galactose to glucosylceramide has also been proven to be involved in the regulation of cell proliferation and angiogenesis (Rajesh et al., 2005). In addition, AFD, an inherited lysosomal storage disorder that frequently results in renal failure, hypertensive cardiac disease, and vascular disease, is linked to UGCG (Stamerra et al., 2021). According to recent research, vascular diseases are primarily caused by globotriaosylceramide (Gb3) accumulation, which results in endothelial cell dysfunction. However, selective UGCG inhibitors can effectively remove Gb3 from AFD cell lines and stop vascular disease progression (Abe et al., 2000).

In this study, after observing that the expression of UGCG in the diseased vascular tissue of VM patients and HUVECs^TIE2-L914F^ significantly increased, we established a stable UGCG-overexpressing HUVECs line and used the UGCG-specific inhibitor Genz to inhibit the expression of UGCG in HUVECs^TIE2-L914F^ for *in vitro* experiments. The findings demonstrated that UGCG overexpression considerably improved the capacity of HUVECs to proliferate, migrate, and form tubes, whereas UGCG inhibition dramatically decreased these same functions in HUVECs^TIE2-L914F^.

Previous studies identified activating somatic mutations in the gene encoding the endothelial-specific tyrosine kinase receptor TEK (TIE2) in almost half of sporadic VM cases, and PIK3CA mutations in approximately 27% of the others (Du et al., 2017; Si et al., 2020). The TIE2 receptor activates the PI3K/AKT/mTOR pathway by binding to its ligands Ang1 and Ang2 (Bilimoria and Singh, 2019). PIK3CA encodes the 110-kD catalytic α-subunit of PI(3)K (p110α), which leads to the activation of PDK1 and phosphorylates AKT on Thr308 (Venot et al., 2018). *In vitro* studies have confirmed that TIE2 and PIK3CA share a common cross-signal transduction pathway, which regulates angiogenesis and stability by affecting the PI3K/AKT/mTOR pathway (Seront et al., 2019).

As previously stated, UGCG is an important enzyme that catalyzes glucosylceramide production, which is linked to the AKT/mTOR pathway. Previous studies have reported that UGCG overexpression stimulates glucosylceramide accumulation in cells, thereby promoting the formation of a range of complex GSLs. This strengthens GSL integration on the cell membrane and changes the lipid composition of the cell membrane, leading to changes in the biophysical membrane properties of GSL-enriched microdomains, thus leading to AKT/mTOR pathway activation (Shayman, 2018; Wu et al., 2018). Studies on emphysema and other related diseases have found that inhibiting UGCG expression can promote apoptosis of human pulmonary microvascular endothelial cells by inhibiting the activation of the mTOR pathway (38). Owing to the important role of the AKT/mTOR pathway in the occurrence and development of VM and the fact that UGCG can affect the activation of the AKT/mTOR pathway, we speculate that UGCG may affect the behavior of vascular endothelial cells by regulating the AKT/mTOR pathway and ultimately participate in the development of VM.

We further evaluated the mechanism of action of UGCG in HUVECs and HUVECs^TIE2-L914F^. As expected, we found that overexpression of UGCG could activate the phosphorylation of AKT and mTOR, and the phosphorylation of AKT and mTOR decreased after UGCG inhibition. These findings suggest that UGCG may promote VM formation through the activation of the AKT/mTOR pathway and that inhibition of UGCG expression may provide a possible therapeutic strategy for the treatment of partial VM.

## Conclusion

This study identified high UGCG expression in VM patients and HUVECs^TIE2-L914F^. By conducting a series of relevant experiments on vascular endothelial cell lines, we demonstrated that UCGC can regulate AKT/mTOR signaling and affect cell proliferation, migration, and tube formation activities, thus providing a potential therapeutic target for the treatment of VM.

## Data Availability

The datasets presented in this study can be found in online repositories. The names of the repository/repositories and accession number(s) can be found in the article/Supplementary Material.
